# Predictors of recurrence in breast cancer patients with a pathologic complete response after neoadjuvant chemotherapy

**DOI:** 10.1038/sj.bjc.6605769

**Published:** 2010-07-06

**Authors:** M Tanioka, C Shimizu, K Yonemori, K Yoshimura, K Tamura, T Kouno, M Ando, N Katsumata, H Tsuda, T Kinoshita, Y Fujiwara

**Affiliations:** 1 Breast and Medical Oncology Division, National Cancer Center Hospital, Tokyo, Japan; 2 Medical Oncology Division, Hyogo Cancer Center, Hyogo, Japan; 3 Department of Clinical Trial Design and Management, Translational Research Center, Kyoto University Hospital, Kyoto, Japan; 4 Division of Diagnostic Pathology, National Cancer Center Hospital, Tokyo, Japan; 5 Division of Breast Surgery, National Cancer Center Hospital, Tokyo, Japan

**Keywords:** breast cancer, pathologic complete response, neoadjuvant chemotherapy, predictive factor, trastuzumab

## Abstract

**Background:**

Although a pathologic complete response (pCR) after neoadjuvant chemotherapy is associated with favourable outcomes, a small proportion of patients with pCR have recurrence. This study was designed to identify factors predictive of recurrence in patients with pCR.

**Methods:**

A total of 449 breast cancer patients received neoadjuvant chemotherapy, and 88 evaluable patients had a pCR, defined as no evidence of invasive carcinoma in the breast at surgery. The clinical stage was II in 61 patients (69%), III in 27 (31%). All patients received taxanes and 92% received anthracyclines. Among 43 patients with HER2-positive tumours, 27 received trastuzumab. Cox regression analyses were performed to identify predictors of recurrence.

**Results:**

Median follow-up was 46.0 months. There were 12 recurrences, including 8 distant metastases. The rate of locoregional recurrence was 10.4% after breast-conserving surgery, as compared with 2.5% after mastectomy. Multivariate analysis revealed that axillary metastases (hazard ratio (HR), 13.6; *P*<0.0001) and HER2-positive disease (HR, 5.0; *P*<0.019) were significant predictors of recurrence. Five of six patients with both factors had recurrence. Inclusion of trastuzumab was not an independent predictor among patients with HER2-positive breast cancer.

**Conclusion:**

Our study results suggest that HER2 status and axillary metastases are independent predictors of recurrence in patients with pCR.

Neoadjuvant chemotherapy is a widely accepted treatment not only for locally advanced breast cancer, but also for earlier-stage operable disease (van der Hage *et al*, 2001; Bonadonna *et al*, 1998; Bear *et al*, 2003). Mauri *et al* (2005) performed a meta-analysis of clinical trials comparing patients who received preoperative chemotherapy with those who received postoperative chemotherapy. Death, disease progression, and distant recurrence were equivalent in both the arms. The main advantages of neoadjuvant chemotherapy included the evaluation of the *in vivo* chemosensitivity of tumours in individual patients; minimisation of micrometastases; and surgical downstaging of tumours, allowing breast-conserving surgery (BCS) to be performed in patients who might have otherwise required a mastectomy. However, the survival advantage of neoadjuvant chemotherapy appears to be negligible (Fisher *et al*, 1997; Bonadonna *et al*, 1998; Kuerer *et al*, 2001; Wolmark *et al*, 2001).

In several studies, a pathologic complete response (pCR), defined as the absence of invasive tumour in the breast only or in the breast and axilla, correlates with a far lower risk of subsequent recurrence, as well as with improved overall survival (Fisher *et al*, 1997, 1998; Bonadonna *et al*, 1998; Morrell *et al*, 1998; Kuerer *et al*, 1999; Chollet *et al*, 2002). Thus, efforts have been made to increase pCR rates by using more effective drugs and treatment regimens (Smith *et al*, 2002; Buzdar *et al*, 2005); the achievement of pCR has become the primary end point of many clinical studies.

Although a pCR is associated with favourable outcomes in most patients, some patients with pCR have disease recurrence. Previous studies have reported 5-year recurrence rates of 13–25% (Fisher *et al*, 1998; Morrell *et al*, 1998; Kuerer *et al*, 2001; Wolmark *et al*, 2001). Only a few studies have examined predictors of recurrence in patients who have a pCR to neoadjuvant treatment (Ring *et al*, 2004; Gonzalez-Angulo *et al*, 2005; Guarneri *et al*, 2006). We therefore retrospectively analysed predictive factors of recurrence in patients with breast cancer who achieved a pCR after neoadjuvant chemotherapy.

## Patients and methods

### Patients

This was a retrospective study of 88 evaluable patients with primary breast carcinoma who had a pCR after receiving neoadjuvant chemotherapy at National Cancer Center Hospital, Tokyo between 1996 and 2006. The follow-up period was completed in December 2008. The locoregional or distant recurrences were evaluated on physical examination, or by radiological imaging.

### Histopathology

All patients were confirmed to have invasive carcinoma histologically by core needle biopsy. Surgical specimens were sectioned at 7- to 10-mm thick slices, and the pathological response was evaluated by pathologists specialised in breast pathology. The histologic type of the primary tumour was classified according to the *General Rules for Clinical and Pathological Recording of Breast Cancer*, The Japanese Breast Cancer Society (2004). The histologic grade of the tumours was classified according to the Elston–Ellis classification system (Elston and Ellis, 1991). The patients' levels of oestrogen receptor (ER, 1D5; Dako, Glostrup, Denmark), progesterone receptor (PgR, 1A6; Novocastra, Newcastle Upon Tyne, UK), and HER2 (HercepTest, Dako) were measured by immunohistochemical (IHC) analysis of paraffin-embedded tissue specimens. Oestrogen receptor and PgR were classified as positive if more than 10% of cancer cell nuclei were stained, regardless of the staining intensity. HER2-positive status was defined as IHC (3+); more than 10% of cancer cells markedly positive, or positive results of fluorescence *in situ* hybridisation (FISH) for HER2 amplification, that is, a HER2/CEP17 signal ratio of 2.0 (Vysis Pathvysion; Abbott, Chicago, IL, USA). IHC (2+) tumours, in which more than 10% of cancer cells were moderately positive, were excluded from the analysis without performing FISH test.

A wide range of criteria have been used to define pCR, and a consensus has yet to be reached. In this study, pCR was defined as no evidence of invasive carcinoma in the breast at the time of surgery in line with the criteria of the National Surgical Adjuvant Breast and Bowel Project B-18 (Wolmark *et al*, 2001) and the recommendations of Sataloff *et al* (1995). Because the presence or absence of residual ductal carcinoma *in situ* (DCIS) after preoperative therapy does not influence long-term rate of local recurrence or overall survival (Mazouni *et al*, 2007), we included patients with residual DCIS in the category of pCR.

### Treatment

Neoadjuvant chemotherapy was indicated in patients with clinical stage II or III primary breast cancer whose tumours were larger than 3 cm. Although the potential benefits of adding taxanes to anthracycline-based regimens remain controversial in terms of long-term outcomes (Bear *et al*, 2006), regimens combining anthracyclines with taxanes, either sequentially or concomitantly, are widely used. In this study, neoadjuvant chemotherapy regimens included (1) four cycles of doxorubicin (DOX, 50 mg m^−2^) and docetaxel (DTX, 60 mg m^−2^) (AT), followed by additional adjuvant treatment with two cycles of AT or four cycles of intravenous cyclophosphamide, methotrexate, and 5-fluorouracil (CMF); (2) four cycles of fluorouracil (500 mg m^−2^)/epirubicin (100 mg m^−2^)/cyclophosphamide (600 mg m^−2^) (FEC) along with 12 weekly cycles of paclitaxel (80 mg m^−2^); (3) four cycles of doxorubicin (60 mg m^−2^)/cyclophosphamide (600 mg m^−2^) (AC) along with 12 weekly cycles of paclitaxel (80 mg m^−2^); (4) twelve weekly cycles of paclitaxel (80 mg m^−2^) only; and (5) four cycles of AC along with four cycles of DTX (60 mg m^−2^). After November 2002, patients with HER2-positive tumours received trastuzumab (initially 4 mg kg^−1^ followed by 2 mg kg^−1^ weekly) in combination with paclitaxel for 12 weeks. Trastuzumab was not administered post-operatively because it had not been approved for use in an adjuvant setting in Japan until 2007.

As for breast surgery, patients underwent either mastectomy (*n*=40) or BCS (*n*=48). Axillary lymph node dissection or sentinel lymph node biopsy alone was additionally performed. The decision to perform BCS was based on the ability to remove residual disease completely with optimal cosmetic results; patient preference was also considered. Twenty-one patients (24%) received adjuvant endocrine therapy including tamoxifen, anastrozole, or both drugs for 5 years if either the pre-treatment biopsy specimen or the surgical specimen obtained after chemotherapy was positive for ER or PgR. We defined surgical margin positive if the tumour cells were directly exposed to the margin.

Postoperative radiotherapy was administered to 60 patients (68%) who had either undergone BCS or had locally advanced disease. The radiotherapy protocol was as follows: after mastectomy, patients with clinical stage III disease received radiotherapy, delivered in 2 Gy fractions to chest wall and axilla (total dose 50 Gy). After BCS, all patients received radiotherapy, delivered in 2 Gy fractions to the breast (total dose 50 Gy). A booster dose was delivered to the tumorectomy bed if the surgical margin was positive. Regardless of the surgical methods, patients with four or more positive axillary lymph nodes received radiotherapy, delivered in 2 Gy fractions to subclavicular region (total dose 50 Gy).

### Clinical significance of locoregional recurrence after neoadjuvant chemotherapy

The impact of locoregional recurrence (LRR) survival after neoadjuvant chemotherapy on survival remains poorly understood. However, patients with LRR after adjuvant chemotherapy, especially those with ER-negative tumours, have substantially worse outcomes regardless of axillary node status (Wapnir *et al*, 2006; Anderson *et al*, 2009). Among patients who achieved a pCR in neoadjuvant setting in our study, the ER-negative rate was 73% and higher than that of patients in adjuvant settings. This suggests the LRR after neoadjuvant chemotherapy might be a negative prognostic factor.

### Statistical analysis

Statistical analyses were performed using SAS, version 9.2 (SAS Institute Inc., Cary, NC, USA). The log-rank test was used to identify predictive factors associated with recurrence after the achievement of pCR. Then, variables with *P*-values of ⩽0.20 on univariate analysis were included in the multivariate models. Multivariate analysis with a Cox proportional-hazards model was used to identify independent predictors in all 88 patients. Models were selected by stepwise forward analysis, retaining variables significant at the *α*=0.05 level for the final model. The Kaplan–Meier product-limit method was used to compute recurrence-free survival according to the number of predictive factors. Recurrence-free survival was measured from the date of initial diagnosis to the date of recurrence (including LRR) or the last follow-up visit. In addition, the relations of recurrence to clinicopathological factors in the 43 patients with HER2-positive tumours were also evaluated. A Cox proportional-hazards model including variables with *P*-values of ⩽0.05 on univariate analysis was used to identify independent predictors of recurrence.

## Results

### Characteristics of patients with relapse

Of 449 patients with breast cancer who received neoadjuvant chemotherapy, 88 (20%) evaluable patients were identified as having a pCR. The median follow-up was 46 months (range, 8–115). Table 1 shows the patient and tumour characteristics. The median age was 54.5 years (range, 29–78). The median diameter of the primary breast tumour was 45.0 mm (range, 25–130). All patients received taxane-based chemotherapy, and 92% also received anthracycline-based therapy.

A total of 12 patients (13.6%) had tumour recurrence (Table 2). All recurrences were diagnosed within 32 months after initial diagnosis. Seven patients died of breast cancer within the follow-up period. Among the six patients who had LRR, five had received BCS as primary surgery, and four had DCIS after neoadjuvant chemotherapy. LRR occurred in 5 of 48 patients (10.4%) after BCS, as compared with only 1 of 40 patients (2.5%) after mastectomy.

### Predictive factors for recurrence in all 88 patients with pCR

The results of univariate analysis of predictive factors for recurrence are shown in Table 3. Variables tested for inclusion in the multivariate model were axillary lymph node metastasis at surgery, HER2 status (positive *vs* negative) and stage (III *vs* II). After controlling for these factors, axillary lymph node metastasis (hazard ratio (HR), 13.6; 95% CI, 4.6–63.3; *P*<0.0001) and HER2-positive disease (HR, 5.0; 95% CI, 1.3–19.3; *P*<0.019) remained significant independent predictors of recurrence (Table 4). According to the number of independent risk factors (HER2-positive disease and axillary lymph node metastasis) for recurrence, the 5-year recurrence-free rate varied between 94.4% for no factor (*n*=36), 89.1% for 1 factor (*n*=46), and 0% for 2 factors (*n*=6).

### Predictive factors for recurrence among 43 patients with HER2-positive disease

Among 43 patients with HER2-positive breast cancer who had a pCR, 27 received trastuzumab. The results of the univariate analysis of predictive factors for recurrence are shown in Table 3. Variables tested for inclusion in the multivariate model were axillary lymph node metastasis at surgery, inclusion of trastuzumab, and stage (III). After controlling for these factors, only axillary lymph node metastasis (HR, 74.6 (8.0–692.9); *P*<0.0001) remained a significant independent predictor of recurrence.

## Discussion

Because a small proportion of patients with breast cancer have recurrence after achievement of a pCR, prediction of the risk of recurrence has an important role in postoperative management. Our multivariate analysis of all 88 patients with a pCR showed that axillary lymph node metastasis and HER2-positive disease were independent predictors of recurrence. Five of the six patients with both of these factors had recurrence after achieving a pCR in our study. Such patients may benefit from additional postoperative therapy and not be optimal candidates for clinical trials with pCR as the primary end point.

Although pCR in this study was defined as no evidence of invasive carcinoma only in the breast, the trial of the University of Texas MD Anderson Cancer Center pCR criteria requires not only complete response of the primary lesion but also the disappearance of axillary metastasis (Green *et al*, 2005). We also performed Cox regression model analysis of 73 patients who satisfied the MD Anderson pCR criteria (results not shown). On univariate analysis, tumour diameter (>50 mm) and grade (3) had *P*-values of ⩽0.20. However, no factor was independently significant in the multivariate analysis. The reasons for the differences in the results according to the definitions of pCR were the smaller sample size, the smaller number of recurrences (only five recurrences), and the elimination of the large influence of axillary lymph nodes on recurrence.

As expected, histopathological lymph node status was a strong predictor of recurrence in patients who had a pCR of their primary tumours. In contrast, HER2 status was found to be a predictor of recurrence for the first time. Gonzalez-Angulo *et al* (2005) studied predictive factors for distant metastasis in 226 patients with pCR. Although HER2 positivity was not a significant predictor of distant metastasis, HER2 status was unknown in 58% of the patients, and only 5% received taxane-based chemotherapy. Interactions between HER2 status and paclitaxel have been reported in an adjuvant setting, especially among patients with ER-negative tumours (Hayes *et al*, 2007). In our exploratory study, HER2 status was assessed by IHC or FISH analyses in all patients, the ER- or PgR-positive rate was low (26%), and all the patients received taxane-based therapy. The combination of these factors may have contributed to the identification of HER2 positivity as a significant independent predictor of recurrence after the achievement of a pCR.

Buzdar *et al* (2005, 2007) and Gianni (2008) reported the results of randomised trials of trastuzumab given with neoadjuvant chemotherapy to patients with HER2-positive breast cancer, and the pCR rate was significantly higher than that in the control arm. However, there are only a few, small randomised trials of neoadjuvant trastuzumab, and so far no study has shown that neoadjuvant trastuzumab can improve overall survival (Rowan, 2009). Indeed, in our study, the pCR rate in patients with HER2-positive breast cancer who received neoadjuvant chemotherapy with trastuzumab was 50% (27 out of 54), which was much higher than that for the study group as a whole (20%, 88 out of 449). However, the inclusion of trastuzumab was not a significant predictor of recurrence on multivariate analysis. This is partly because trastuzumab was not administered post-operatively. The optimal duration of trastuzumab in neoadjuvant and adjuvant setting should be confirmed prospectively in randomised trials.

The demand for BCS is expected to rise as the reported rate of pCR after BCS increases. However, LRR rates after BCS in patients who received neoadjuvant chemotherapy in previous studies have varied from 2.6 to 22.6% (Mauriac *et al*, 1999; Rouzier *et al*, 2001; Peintinger *et al*, 2006). This wide variability has led to uncertainty, and the benefits of BCS have been questioned. Objective evaluation of the safety and effectiveness of BCS has been precluded by the small numbers of patients who have achieved a pCR, different criteria for determining whether BCS is indicated, and different treatment regimens. Mauri *et al* (2005) performed a meta-analysis of clinical trials comparing preoperative with postoperative chemotherapy. Although the proportion of patients with distant recurrence was equivalent in both arms, LRR was more frequent in the preoperative chemotherapy arm, with an HR of about 1.2. In our study, most cases of LRR occurred after BCS, and the proportion of patients with LRR was 10.4% after BCS, as compared with only 2.5% after mastectomy. Our study results suggest that even after achieving a pCR, patients should be carefully followed up for LRR after BCS.

This study was retrospective and lacked a sufficient number of patients with recurrence after the achievement of a pCR to allow us to make firm recommendations for a given treatment option. Despite these limitations, some tentative conclusions can be drawn. First, our retrospective analysis showed that HER2-positive disease and axillary metastasis were independent predictors of recurrence after the achievement of a pCR at the primary site in response to neoadjuvant chemotherapy. This finding suggests that patients with HER2-positive disease and axillary metastasis may be candidates for more aggressive adjuvant therapy even after the achievement of a pCR, but this assumption must be confirmed in future clinical trials. Second, the inclusion of trastuzumab in regimens for neoadjuvant chemotherapy might not be predictive of recurrence, even though the rate of pCR among patients who received trastuzumab was much higher than that among all patients who received neoadjuvant chemotherapy. Third, the rate of LRR was higher after BCS than after mastectomy. Patients who undergo BCS should thus be closely followed up for LRR.

## Figures and Tables

**Table 1:** Patient characteristics

	**All patients (*N*=88)**
**Characteristic**	**No. of patients**
*Age, years*	
⩽50/>50	33/55
	
*Clinical stage*	
II/IIIA/IIIB,IIIC	61/18/9
	
*Pre-treatment pathology*	
Invasive ductal/lobular/mucinous/others	85/1/1/1
	
*Nuclear grade*	
1/2/3/unknown	2/24/61/1
	
*Hormone receptor status*	
ER or PgR positive/both negative	23/65
	
*HER2 status*	
Positive/Negative	43/45
	
*Neoadjuvant chemotherapy*	
FEC → weekly paclitaxel (±trastuzumab)	31 (16 with trastuzumab)
AC → weekly paclitaxel (±trastuzumab)	30 (8 with trastuzumab)
AT (doxorubicin + docetaxel)	19
Weekly paclitaxel (± trastuzumab)	7 (3 with trastuzumab)
AC → docetaxel	1
	
*Surgery*	
Mastectomy/Breast-conserving surgery	40/48

Abbreviations: FEC=fluorouracil+epirubicin+cyclophosphamide; AC=doxorubicin+cyclophosphamide; PgR=progesterone receptor.

**Table 2:** Characteristics of patients with recurrence

		**Initial diagnosis**			**Operative information**			**State at recurrence**			
**No.**	**Age**	**Tumour diameter**	**HER2**	**ER or PgR**	**Ax. M.**	**DCIS**	**BCS**	**LRR**	**Distant M.**	**Brain M.**	**RFS**
1	39	90	−	−	−	−	−	−	+	+	8
2	33	52	−	+	−	−	+	−	+	−	26
3	62	55	+	−	−	−	+	+	+	−	26
4	29	35	+	+	−	+	+	+	+	−	30
5	58	42	+	−	−	−	+	+	+	−	32
6	55	65	+	−	+	+	−	−	+	−	32
7	63	49	+	−	+	+	−	+	−	−	18
8	36	34	−	+	+	-	−	−	+	−	20
9	49	30	+	−	+	+	−	−	+	−	21
10	56	25	+	−	+	+	+	+	−	−	21
11	50	55	+	−	+	-	+	−	+	+	29
12	71	60	−	−	+	+	+	+	−	−	32

Abbreviations: Ax. M.=axillary lymph node metastasis; M.=metastasis; BCS=breast-conserving surgery; RFS=recurrence-free survival (months); LRR=locoregional recurrence; ER=oestrogen receptor; PgR=progesterone receptor; HER2=human epidermal growth factor 2; DCIS=ductal carcinoma *in situ*.

**Table 3:** Univariate analysis of predictive factors for recurrence

	**All patients (*N*=88)**			**HER2 positive (*N*=43)**		
**Characteristic**	**No.**	**Patients with recurrence (%)**	***P*-value**	**No.**	**Patients with recurrence (%)**	***P*-value**
*Age*						
>50 years old	55	10.9		28	17.9	
⩽50 years old	33	18.2	0.28	15	20	0.83
						
*Tumour diameter*						
> 50 mm	30	20.0		12	25.0	
⩽50 mm	58	10.3	0.22	31	16.1	0.44
						
*Clinical stage*						
II	61	9.8		30	13.3	
III	27	22.2	0.09	13	30.8	0.11
						
*ER or PgR*						
Positive	23	13.0		9	11.1	
Negative	65	13.8	0.87	34	20.6	0.45
						
*HER2*						
Positive	43	18.6				
Negative	45	9.1	0.19			
						
*Nuclear grade*						
3	61	14.5		28	21.4	
1–2	26	11.5	0.71	15	13.3	0.49
						
*Type of chemotherapy*						
Anthracycline + taxane	81	13.4		39	18.0	
Taxane based	7	28.6	0.38	4	25.0	0.91
						
*Type of chemotherapy*						
With trastuzumab	27	7.4		27	7.4	
Without trastuzumab	61	16.4	0.28	16	37.5	0.015
						
*Surgery*						
Mastectomy	40	12.5		21	23.8	
BCS	48	14.6	0.84	23	13.6	0.48
						
*Residual DCIS*						
Present	39	15.4		23	21.7	
None	49	12.2	0.65	20	15.0	0.50
						
*No. of LNs examined*						
⩽10	15	14.7		7	14.3	
>10	73	13.7	0.93	36	19.4	0.79
						
*Axillary LN status*						
Node positive	15	46.7		6	83.3	
Node negative	73	6.9	<0.001	37	8.1	<0.001

Abbreviations: ER=oestrogen receptor; PgR=progesterone receptor; HER2=human epidermal growth factor receptor 2; pCR=pathological complete response; BCS=breast-conserving surgery; DCIS=ductal carcinoma *in situ*; LN=lymph node.

**Table 4:** Multivariate analysis of predictors of recurrence (all 88 patients)

**Characteristic**	**HR**	***P*-value**	**95% CI**
Axillary lymph node metastasis	13.6	<0.0001	4.6–63.3
HER2-positive disease	5.0	0.019	1.3–19.3

Abbreviations: HR=hazard ratio; CI=confidence interval; HER2=human epidermal growth factor receptor 2.
